# Hand–foot syndrome in sorafenib and lenvatinib treatment for advanced thyroid cancer

**DOI:** 10.1530/ETJ-24-0009

**Published:** 2024-07-29

**Authors:** Elisa Minaldi, Virginia Cappagli, Loredana Lorusso, Laura Valerio, Carlotta Giani, Matilde Viglione, Laura Agate, Eleonora Molinaro, Antonio Matrone, Rossella Elisei

**Affiliations:** 1 Department of Clinical and Experimental Medicine, Unit of Endocrinology, University Hospital of Pisa, Via Paradisa, Pisa, Italy

**Keywords:** hand–foot syndrome, lenvatinib, sorafenib, thyroid cancer, tyrosine kinase inhibitors

## Abstract

**Objective:**

The aim of this study was to assess the clinical impact of hand–foot syndrome (HFS) during treatment with two multikinase inhibitors, sorafenib and lenvatinib, in a large group of patients with advanced thyroid cancer. Moreover, we looked for possible associations between HFS occurrence and clinical and pathological features.

**Methods:**

We retrospectively evaluated 239 patients with advanced thyroid cancer: 165 treated with lenvatinib and 74 with sorafenib. Statistical analyses were performed to verify which features could be correlated with HFS development.

**Results:**

HFS was observed in 35/74 (47.4%) and in 43/165 (26.7%) patients treated with sorafenib or lenvatinib, respectively. The median latency from the drug beginning and HFS appearance was 27 days for sorafenib and 2.9 months for lenvatinib. G3/G4 toxicity was observed in 16/35 (45.7%) patients treated with sorafenib and only in 3/43 (7%) treated with lenvatinib. Drug dose reduction due to HFS was required in 19/74 (25.7%) and 3/165 (1.8%) patients treated with sorafenib and lenvatinib, respectively. HFS occurrence was significantly associated with a longer duration of therapy in both groups.

**Conclusion:**

HFS was a frequent adverse event during both lenvatinib and sorafenib therapy, with a higher frequency and toxicity grade during sorafenib treatment. HFS was the most frequent reason for drug reduction or discontinuation in patient treated with sorafenib. Early diagnosis of HFS is important to allow early intervention, possibly in a multidisciplinary setting, and to avoid treatment discontinuation, which is highly relevant to obtain the maximum effectiveness of systemic therapy.

## Introduction

Thanks to the results of DECISION and SELECT trials, sorafenib and lenvatinib are the only MKIs approved by both the U.S. Food and Drug Administration (FDA) and the European Medical Agency (EMA) for the first-line treatment of advanced radioiodine (RAI)-refractory DTC (1, 2). The molecular targets are almost completely shared by the two compounds: in fact, both are small oral molecules with a strong anti-angiogenic activity due to the inhibition of VEGF1–3, EGF, PDGF, FGF, KIT, RET receptors and, in case of sorafenib, also RAF (3).

The anti-tumoral activity of these MKIs is based on a cytostatic effect on tumoral cells, causing the rapid shrinkage of tumoral mass through the inhibition of tumoral angiogenesis, but the activity of these drugs against other receptors may be unfortunately responsible for their ‘off-targeted’ activity, leading to the appearance of side effects/adverse events (AEs), shared, in the majority of cases, by all types of molecules (3). The management of AEs is crucial both to optimize patients’ compliance and to avoid potentially life-threatening consequences and it should be tailored on each patient (4, 5, 6). One of the most frequent AEs occurred both in the DECISION and SELECT trial was the dermatological toxicity, mainly represented by hand–foot syndrome (HFS). This was defined as minimal skin changes or dermatitis of hands and feet evolving with hyperkeratosis, desquamation and ulceration of the palmar surfaces of hands or plantar surface of feet, associated with significant impairment of patient’s quality of life, and in some cases bleeding and/or pain (7). According to the data reported in clinical trials, it occurred in 76.3% of patients treated with sorafenib (1) and 31.8% in those treated with lenvatinib (2).

The aim of this study was to assess the prevalence and the clinical impact of HFS in the real-world clinical practice in a large group of patients diagnosed and followed up at our department and treated with lenvatinib or sorafenib. Moreover, we looked for possible associations between the occurrence of HFS and clinical, pathological and radiological features.

## Patients and methods

### Patients

We retrospectively evaluated the epidemiological, pathological and clinical data of a cohort of 239 patients with advanced TC, requiring systemic therapy with MKIs, followed from 2012 to 2022 at the Endocrine Unit of a tertiary referral center for thyroid diseases. According to the administered drug, we divided patients in two groups, named lenvatinib and sorafenib group, composed by 165 and 74 patients, respectively.

Patients were treated with lenvatinib within the clinical trials (SELECT trial-NCT01321554 and Expanded Access Program study-EAP) (2, 5), or as ‘compassionate use’ or with the commercial drug after its approval in 2016.

Patients were treated with sorafenib within the DECISION clinical trial (NCT00984282) (1) or as ‘off label’ compassionate use. This last approach was used before the official drug approval when no other drugs were available, as second or third-line therapy after chemotherapy, radiotherapy or other MKIs.

Patients enrolled in the registrative clinical trials had to respect more strict and specific inclusion criteria (1, 2), compared to those treated as ‘compassionate use’ or after the drug approval. To start the drug, all patients had to present disease progression or a large and symptomatic tumor burden, while significant cardiovascular, hematopoietic, hepatic and renal failure represented exclusion criteria. Outside of clinical trials, the Eastern Cooperative Oncology Group Performance Status (ECOG PS) score could be up to 2, and there were no limitations on previous oncological treatments, such as other MKIs.

All patients signed an informed consent before starting the therapies, reporting all the AEs that can be developed during the treatment. As for policy of our hospital, all patients gave signed informed consent for the use of all their clinical, biochemical and pathological data collected during treatment for research purposes. The study was approved by the local ethical committee (Comitato Etico Area Vasta Nord Ovest – CEAVNO).

### Treatment management and follow-up

In all patients enrolled in the SELECT and EAP studies the initial dose of lenvatinib was 24 mg daily (2, 5), while in patients treated with lenvatinib as ‘compassionate use’ or after its approval, the starting dose was 24 mg/day or lower, according to the patient's clinical condition and comorbidities and doctors’ judgment. Similarly, in the sorafenib group, all patients enrolled in DECISION clinical trial started sorafenib at the dose of 800 mg/day (1), while lower doses were administered in case of ‘off-label’ use of sorafenib.

All patients were evaluated at the moment of the screening and then every 3–6 months, until the evidence of disease’s progression and/or events as death or serious AEs requiring permanent discontinuation of the drug. At each follow-up visit, clinical assessment of patients, routine blood samples and biochemical tumoral markers evaluation were performed. CT scan with contrast injection was performed at the screening visit and then every 3–6 months.

During follow-up, drug dose reduction or transient interruption was indicated in case of severe AEs, graded according to the Common Terminology Criteria of Adverse Events (CTCAE) (8). The treatment was definitively discontinued in case of progression according to the Response Evaluation Criteria in Solid Tumors (RECIST) criteria (9) or in case of severe AEs.

### Statistical analysis

Categorical variables were compared by *χ*^2^ test, while normally distributed continuous variables were compared using Student’s *t*-test, based upon variance assessment by Levene test. Nonparametric continuous variables were compared by Mann–Whitney *U* test. Pearson’s correlation coefficient was used to measure the association between continuous variables. Statistical significance level was set at *P* < 0.05. Data analyses were performed using SPSS software (IBM SPSS Statistics, version 25).

## Results

### Descriptive analysis

All the demographic, clinical and pathological features of the two groups of patients are shown in Table 1.

**Table 1:** Clinical and pathological data of two groups of patients with advanced thyroid cancer treated with sorafenib and lenvatinib, respectively. Data are presented as *n* (%), mean ± s.d. or as median (IQR).

Variables	Sorafenib (*n* = 74)	Lenvatinib (*n* = 165)
Sex		
Females	40 (54.1)	82 (49.7)
Males	34 (45.9)	83 (50.3)
Age at initiation of MKI therapy, years		
Mean ± s.d.	61.9 ± 10.9	65.64 ± 10.00
Range	35–84	36–89
TC histotypes		
Papillary	21 (28.4)	89 (53.9)
Follicular	21 (28.4)	34 (20.6)
Oncocytic	0	9 (5.5)
Poorly differentiated	10 (13.5)	30 (18.2)
Anaplastic	22 (29.7)	3 (1.8)
T stage at diagnosis		
Tx	15 (20.3)	30 (18.2)
T1	2 (2.7)	9 (5.5)
T2	3 (4.1)	30 (18.2)
T3	21 (28.4)	61 (37)
T4	33 (44.6)	35 (21.2)
N stage at diagnosis		
Nx	1 (1.4)	7 (4.2)
N0	33 (44.6)	68 (41.2)
N1	40 (54.1)	90 (54.6)
M stage at diagnosis		
Mx	2 (2.7)	13 (7.9)
M0	38 (51.4)	92 (55.8)
M1	34 (45.9)	60 (36.3)
8th edition AJCC staging		
I	15 (20.3)	42 (26.4)
II	8 (10.8)	36 (22.6)
III	7 (9.5)	18 (11.3)
IVA	8 (10.8)	14 (8.8)
IVB	28 (37.8)	49 (29.7)
IVC	8 (10.8)	–
ECOG PS at MKI start		
0-1	59 (79.7)	72 (59)
2-3	15 (20.3)	50 (41)
Previous treatments		
131-I	49 (66.2)	148 (89.7)
Chemotherapy	30 (40.5)	17 (10.3)
External radiotherapy	33 (44.6)	74 (45.1)
Other MKIs	7 (9.5)	19 (11.5)
None	9 (12.2)	10 (6.1)
Duration of MKI therapy (months)	5.01 (0.4–91)	15 (6.16–28.75)
Starting dose of MKI (mg/day)	800	24 (14–24)

131-I, radioiodine; AJCC, American Joint Committee on Cancer; ECOG PS, Eastern Cooperative Oncology Group Performance Status; IQR, interquartile range; MKI, multikinase inhibitors; TC, thyroid carcinoma.

### Sorafenib

The group treated with sorafenib was represented by 74 patients, of which 40/74 (54.1%) were females and 34/74 (45.9%) were males. The mean age at the starting of sorafenib therapy was 61.9 ± 10.9 years (range: 35–84). The most frequent TC histotype was anaplastic thyroid carcinoma (ATC), diagnosed in 22/74 patients (29.7%), followed by papillary thyroid carcinoma (PTC) in 21/74 patients (28.4%), follicular thyroid carcinoma (FTC) in 21/74 (28.4%) and PDTC in 10 (13.5%).

Most patients had an advanced stage of disease at diagnosis and more than half had also distant metastases. In particular, T3/T4 stage was found in 54/74 patients (73%), N1 stage in 40/74 (54.1%) and M1 stage in 34/74 patients (45.9%). Regarding previous TC treatments, 49/74 (66.2%) received 131-I treatments before starting sorafenib, while 33/74 (44.6%) were submitted to other single systemic (chemotherapy, other MKI) or local treatments (i.e. external beam radiotherapy) and 18/74 (24.3%) to multiple and subsequent treatments: particularly 30/74 (40.5%) patients received chemotherapy, 33/74 (44.6%) external beam radiotherapy and 7/74 (9.55) other MKIs, and only 9/74 patients (12.2%) were directly treated with sorafenib after surgery.

The duration of sorafenib therapy was extremely variable and the median was 5 months (IQR: 0.4–91). More in details, 7/74 patients (9.5%%) took the drug for < 1 month, 39/74 patients (52.7%) from 1 to 6 months, 9/74 (12.2%) from 6 to 12 months and 19/74 (25.7%) more than 1 year.

The epidemiological, pathological and clinical features of subgroup of patients clustered according to the duration of treatment are reported in Supplementary Table 1 (see section on supplementary materials given at the end of this article).

The starting dose of sorafenib was 800 mg/day in almost all patients (69/74 patients, 93.2%), while in 3/74 (4.1%) and 2/74 (2.7%) was respectively 400 and 600 mg/day. During follow-up in 46/74 cases (62.2%%) a dose reduction was required due to AEs.

### Lenvatinib

The group treated with lenvatinib consisted of 165 patients, 82 females (49.7%) and 83 males (50.3%). The mean age at the starting of lenvatinib was 65.64 ± 10 years (range: 36–89). The distribution of histotypes was as follows: PTC in 89/165 cases (53.9%), FTC in 34/165 cases (20.6%), PDTC in 30/165 cases (18.2%), Oncocytic TC in 9/165 (5.5%), ATC 3/165 (1.8%). Regarding tumor stage, T3/T4 stage was found in 96 patients (58.2%), N1 stage in 90 patients (54.6%) and M1 stage in 60 patients (36.3%). Before starting lenvatinib, radioiodine treatments only were performed in 71 patients (43%), while 84 (50.9%) received in addition other systemic (chemotherapy, other MKI) or local treatments (i.e. external beam radiotherapy) (Table 1): particularly 17/165 (10.3%) patients received chemotherapy, 74/165 (45.1%) external beam radiotherapy and 19/165 (11.5%) other MKIs, only 10/165 patients (6.1%) were directly treated with lenvatinib after surgery.

The median duration of lenvatinib therapy was 15 months (IQR: 6.16–28.75). In particular, 7/165 patients (4.2%) assumed the drug for < 1 month, 34/165 patients (20.6%) from 1 to 6 months, 33/165 (20%) from 6 to 12 months, 91/165 (55.2%) for more than 1 year. The epidemiological, pathological and clinical features of subgroups of patients clustered according to the duration of treatment are reported in Supplementary Table 1.

The median starting dose of Lenvatinib was 24 mg/day (IQR: 14–24; range: 4–24).

### Hand–foot syndrome prevalence and associations

#### Sorafenib

**Figure 1:**
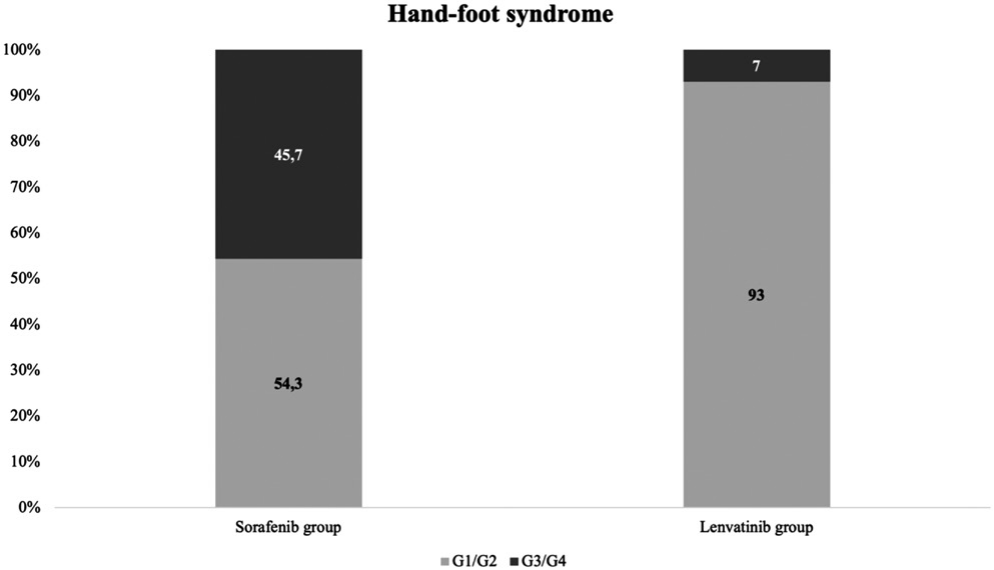
Distribution of severity of hand–foot syndrome (graded according to the Common Terminology Criteria of Adverse Events) in two groups of patients with advanced thyroid cancer treated with sorafenib and lenvatinib.

In the sorafenib group HFS was observed in 35/74 cases (47.4%). In particular G1/G2 toxicity was observed in 19/35 patients (54.3%) and G3/G4 in the remaining 16/35 ones (45.7%) (Fig. 1). In particular, 7/35 (20%) patients experienced HFS alone without any other AEs, while in the remaining 28/35 (80%) other toxicities were reported during follow-up, as shown in Table 2. Nineteen out of 74 (25.7%) had to reduce the drug dose due to this AE. In the remaining 27/74 (36.4%) patients other AEs was responsible for the dose reduction: in 7/27 (9.5%) for asthenia/anorexia, in 6/27 (8.1%) for mucositis, in 4/27 (5.4%) for other comorbidities, in 4/27 (5.4%) for laboratory abnormalities, in 4/27 (5.4%) for cutaneous rash and in 2/27 (2.7%) for gastrointestinal toxicity. In 28/74 (37.8%) patients no drug reduction was needed. No patients experienced a complete recovery from HFS after drug withdrawal or dose reduction, but in most cases the toxicity grade improved at G1/G2 grade and did not impact on patient quality of life and drug compliance. There were no patients who experienced a HFS relapse or worsening during follow-up.

**Table 2:** Prevalence of other adverse events in patients with hand–foot syndrome (HFS). Data are presented as *n* (%).

Adverse events	Sorafenib (*n* = 28^a^)	Lenvatinib (*n* = 43)
Hypertension	5 (17.9)	35 (81.4)
Anorexia/weight loss	6 (21.4)	29 (67.4)
Diarrhea	5 (17.9)	19 (44.2)
Nausea/vomiting	1 (3.6)	23 (53.5)
Mucositis	12 (42.9)	28 (65.1)
Asthenia	9 (32.1)	33 (76.7)
Cutaneous rash	8 (28.6)	–
Fistula	2 (7.1)	8 (18.6)
Hyposurrenalism	–	5 (11.6)
Cholecystitis	–	7 (16.3)

^a^7/35 patients showed only HFS and are not included in this table.

The median latency period observed between the diagnosis of HFS and the starting of sorafenib was 27 days (IQR: 63; range: 7–150 days).

When we correlated the presence of HFS with the clinical and pathological features, some statistically significant associations were found, as reported in Table 3. A statistically significant higher prevalence of HFS was found in patients with longer duration therapy (*P* = 0.01) (median 9.1 vs 3.3 months). Instead, no correlations were found with sex, age at the starting of sorafenib therapy, N an M stage, other previous treatments, TC histotype and first radiological evaluation.

**Table 3:** Correlation between hand–foot syndrome occurrence and clinicopathological features in two groups of patients with advanced thyroid cancer treated with sorafenib and lenvatinib, respectively. Data are presented as mean ± s.d., *n* (%) or as median (IQR).

	Sorafenib group (*n* = 74)			Lenvatinib group (*n* = 165 )		
	HFS (*n* = 35)	No-HFS (*n* = 39)	*P*	HFS (*n* = 43)	No-HFS (*n* = 122)	*P*
Age at MKI start (years)	61.4 ± 10.7	62.2 ± 11.1	0.76	63.00 ± 8.97	66.61 ± 10.14	0.03
Gender			0.16			0.39
Female	22 (55)	18 (45)		24 (29.6)	57 (70.4)	
Male	13 (38.2)	21 (61.8)		9 (12.9)	61 (87.1)	
MKI therapy duration (months)	9.1 (3.3–26)	3.3 (1.3–6.3)	0.01	21 (12–48)	12.58 (4.5–25)	0.001
ECOG PS			0.18			0.157
0–1	30 (50.8)	29 (49.2)		25 (34.7)	47 (65.3)	
2–3	5 (33.3)	10 (66.7)		9 (21.9)	41 (78.1)	
Prior treatments other than 131-I^a^			0.55			0.45
Monotherapy	16 (48.5)	17 (51.5)		17 (20.2)	67 (78.8)	
Multitherapy	11 (61.1)	7 (38.9)		1 (8.3)	11 (91.7)	
Previous treatment with other MKI			0.7			0.08
First line	31 (46.3)	36 (53.7)		41 (28.1)	105 (71.9)	
Second line	4 (57.1)	3 (42.9)		2 (10.5)	17 (89.5)	
Starting dose of MKI (mg/day)	800 (0)	800 (0)	0.50	24 (18–24)	24 (14–24)	0.18
Histotypes			0.46			0.49
Papillary	9 (42.8)	12 (57.2)		23 (25.8)	66 (74.2)	
Follicular	13 (61.9)	8 (38.1)		10 (29.4)	24 (70.6)	
Poorly differentiated	4 (40)	6 (60)		6 (20)	24 (80)	
Oncocytic	–	–		4 (44.4)	5 (55.6)	
Anaplastic	9 (40.9)	13 (59.1)		0 (0)	3 (100)	
Distant metastases^b^			0.09			0.25
M0	14 (36.8)	24 (63.2)		26 (28.3)	66 (71.7)	
M1	20 (58.8)	14 (41.2)		12 (31.6)	48 (42.1)	
Lymph node metastases^b^			0.6			0.41
N0	14 (42.3)	19 (57.7)		15 (22.1)	53 (77.9)	
N1	20 (50)	20 (50)		25 (27.8)	65 (72.2)	
First radiological evaluation^c^			0.46			0.56
Partial response + stable disease	31 (54.4)	26 (45.6)		40 (30.1)	93 (69.9)	
Progressive disease	3 (37.5)	5 (62.5)		3 (18.8)	13 (81.2)	

^a^Patients who received no prior thyroid cancer treatment or only 131-I were excluded from the analysis; ^b^Tx, Nx and Mx patients in both groups were excluded from the analysis; ^c^8/74 of sorafenib group and 16/165 of lenvatinib group did not perform any radiological evaluation after drug start.

131-I, radioiodine; ECOG PS, Eastern Cooperative Oncology Group Performance Status; HFS, hand–foot syndrome; IQR, interquartile range; MKI, multikinase inhibitor.

#### Lenvatinib

Forty-three out of 165 (26.7%) developed HFS during lenvatinib treatment. Of these 43 cases of HFS, 24/43 (93%) were classified as G1/G2 and 3/43 (7%) as G3/4 (Fig. 1). In all patients, HFS occurred together with other AEs and their prevalence is reported in Table 2. In 3/165 (1.8 %) patients a drug dose reduction or permanent discontinuation of lenvatinib was necessary due to HFS. In 125/165 patients (75.7%) other AEs were responsible for the first dose reduction: in particular, 42/125 (33.6%) reduced for asthenia/anorexia, in 15/125 (12%) for gastrointestinal toxicity, in 14/125 (11.2%) for laboratory abnormalities, in 12/125 (9.6%) for high blood pressure, in 11/125 (8.8%) for mucositis, in 6/125 cases for fistulization (4.8%), in 4/125 for cholecystitis/cholangitis (3.2%), in 3/125 (2.4%) for cutaneous ulcers and in 18/125 (14.4%) for other reasons. In 37/165 (22.4%) patients, no drug dose change was required.

In 37/43 cases with known information about HFS follow-up, 25/37 (67.5%) had an improvement of HFS due to topical care or drug reduction, followed by a stability overtime but without a complete remission of the AE. On the contrary, in 12/37 (32.5%) patients HFS went in remission after a median time of 9 months (IQR: 4.75–22.5). There were no patients who experienced a HFS relapse or worsening during follow-up.

The median latency period from the drug beginning and HFS was of 2.9 months (IQR: 1.14-6.79; range: 0.16–46.46 months). The occurrence of HFS in patients treated with lenvatinib was significantly associated with younger age (mean: 63.00 ± 8.97 vs 66.61 ± 10.14, *P* = 0.03) and longer duration of lenvatinib therapy (median: 21 months (IQR: 12–48) vs median 12.58 months (IQR: 4.525); *P* = 0.001). Moreover, age and duration of lenvatinib therapy were inversely correlated (Pearson *r*(163) = −0.16; *P* = 0.036).

No significant association was found for sex, presence of distant metastasis, TC histotype, ECOG score, the starting dose of lenvatinib, prior treatments and first radiological evaluation. All the results were summarized in Table 3.

#### Comparing our results with literature

The present study, reporting our experience with both drugs, either in conventional trials or in real life, showed that HFS was a frequent AE, especially during sorafenib (47.4%) respect to lenvatinib therapy (26.7%). Some differences were present between our data and other reported in the literature, as reported in Table 4.

**Table 4:** Frequency and grading of hand-foot syndrome in clinical studies available in literature.

	Study design	TC histotypes in patients, *n*								HFS		Drug
		DTC	MTC	ATC	PTC	FTC	PDTC	OCA	HTC	All grades	Grade≥3	
Capdevila *et al.* (10)	RLMS	16	15	3						21/34 (61%)	8/34 (23.5%)	Sorafenib
Ahmed *et al.* (11)	Phase II CS	19	15							27/34 (79.4%)	15/34 (44.1%)	Sorafenib
Shah *et al.* (12)	Phase II CS			4	41	11				35/56 (62.5%)	4/56 (7.1%)	Sorafenib
Hoftijzer *et al.* (13)	Phase II PSCSA	31								22/31 (71%)	7/31 (22.6%)	Sorafenib
Lam *et al.* (14)	Phase II CS		21							19/21 (90.5%)	3/21 (14.3%)	Sorafenib
Gupta-Abramson *et al.* (15)	Phase II CS		2	1	27					28/30 (93.3%)	3/30 (10%)	Sorafenib
Cabanillas et al (16)	SC off-label use				8	7				9/13 (69.2%)	Not reported	Sorafenib
Brose *et al.* (1)	Phase III DBR trial	207*								158/207 (76.3%)*	42/207 (20.3%)*	Sorafenib
Kim *et al.* (17)	RMS				35	9	3	1		42/48 (87.5%)	32/48 (66.7%)	Sorafenib
Present study	RLMS				21	21	10	22		35/74 (47.4%)	16/35 (45.7%)	Sorafenib
Schlumberger *et al.* (2)	Phase III, DBR-MC				132*	53*	28*		48*	83/261 (31.8%)*	9/261 (3.4%)*	Lenvatinib
Nervo *et al.* (21)	RMS				4	2	6			11/12 (91.7%)	2/12 (16.7%)	Lenvatinib
Berdelou *et al.* (22)	RMS				32	22	19	2		21/75 (28%)	0/75 (0%)	Lenvatinib
Balmelli *et al.* (20)	RMS	13								1/13 (7.7%)	0/13 (0%)	Lenvatinib
Kim *et al.* (17)	RMS				14	7	2			13/23 (56.5%)	7/23 (30.4%)	Lenvatinib
Giani *et al.* (5)	OOLMCS				27	14				12/36 (33.3%)	1/36 (2.8%)	Lenvatinib
Aydemirli *et al.* (23)	RMS				15	9		15		10/39 (26%)	0/39 (0%)	Lenvatinib
Porcelli *et al.* (18)	RMS				4	6	7		6	8/23 (34.8%)	0/23 (0%)	Lenvatinib
De Leo *et al.* (31)	RMS				4	7	2			1/13 (7.7%)	0/13 (0%)	Lenvatinib
Kim *et al.* (19)	RMCCS				42 +1**	4	9			20/56 (36%)	2/56 (4%)	Lenvatinib
Present study	RLMS			3	89	34	30	9		43/165 (26.7%)	3/43 (7%)	Lenvatinib

ATC, anaplastic thyroid carcinoma; CS, clinical study; DBR, double-blind randomized; DTC, differentiated thyroid carcinoma; FTC, follicular thyroid carcinoma; HFS, hand-foot syndrome; MC, multi-centre; MTC, medullary thyroid carcinoma; OCA, oncocytic thyroid carcinoma; OOLMCS, observational open-label multi-center study; PDTC, poorly differentiated thyroid carcinoma; PSCSA, prospective single centre single arm; PTC, papillary thyroid carcinoma; RLMS, retrospective longitudinal monocentre study; RMS, retrospective moncentrer study; RMCS, retrospective multi-centre study; RMCCS, retrospective multi-centre cohort study; SC, single centre. *Considering only patient treated with the drug, excluding those in placebo arm; **1 PTC and FTC.

Regarding sorafenib group, the observed frequency for this AE in our series (47.3%) was lower than others reported, both in the registrative trial (76.3%) (1) and in other single/multicenter studies (varying from 61% to 93.3%) (10, 11, 12, 13, 14, 15, 16, 17). On the contrary, a higher severity of HFS was reported in our study compared to others. In fact in our series the percentage of G3/G4 toxicity was 45.7%, while in the majority of other studies the reported frequency of these grades varied from 10 % to 23.5%. Only in two studies the observed G3/G4 toxicity was consistent with our data, ranging from 44.1% to 66.7% (11, 17). Despite this difference in the degree of toxicity, both in our series and in others, including DECISION trial, the HFS was the main reason for dose reduction.

Regarding lenvatinib group, the prevalence of HFS in our patients (26.7%) is quite consistent with data both from the registrative SELECT trial (31.8%) and from some other real-life multicentric studies, reporting a frequency of HFS, varying from 24.5% to 33.3% (5, 18, 19, 20, 21, 22, 23). On the contrary, only few and smaller single-center studies found different frequency range, from 8% to 91.7%. Regarding the severity of the AE, in our study only 3/43 (7%) patients had severe HFS (grade 3 and 4), quite consistently with the rates observed in the registrative trial of lenvatinib (3.4%) (2) and the Italian multicenter study (2.8%) (5). In other smaller studies a wide range of frequencies have been reported, from 0% to 30.4%. Of these, the two aforementioned studies that reported high rates of HFS, also documented the highest frequency of severe cases (grade > 3): 2/12 (16.7%) (21) and 7/23 (30.4%) (17), respectively.

## Discussion

Among the toxicities of multikinase inhibitors, particularly lenvatinib and sorafenib, HFS is one of the most frequent and it may present with a painful erythema, edema and palm–plantar desquamation, which may lead to an impaired quality of life (5).

The cutaneous toxicity profile seems to be related to the action of MKIs on EGFR, which appears to be the most important mediator of the molecular mechanisms underlying these AEs, in particular the cutaneous rash. The inhibition of EGFR, in fact, in basal keratinocytes induces several alterations of keratinocyte survival, proliferation, differentiation, migration, and attachment and causes an inflammatory reaction (24). On the other hand, the suggested pathogenetic mechanism for the HFS is instead the combined inhibition of VEGFR and PDGFR, which seems to impair the vascular repair mechanisms in high-pressure areas in the body (24). Since the inhibition of either VEGFR or PDGFR alone is not associated with HFS, the combined inhibition of both targets seems to be a crucial event for this toxicity (24). These different molecular mechanisms underlying skin toxicities could explain the different percentage and type of cutaneous AEs during different MKIs treatments. In fact, cutaneous rash is a very frequent AE during vandetanib therapy, since it is a potent inhibitor of EGFR, differently from sorafenib or lenvatinib (Supplementary Table 2) (25, 26, 27, 28, 29, 30). On the contrary, HFS is not observed under vandetanib, that does not inhibits PDGFR, but occurs very frequently under sorafenib and lenvatinib treatment because of their ability to inhibit both VEGFR and PDGFR (Supplementary Table 2) (25, 26, 27, 28, 29, 30). Furthermore, recent data suggested a possible drug-induced reactive oxygen species (ROS) formation which could lead to a keratinocyte damage in the skin and subsequent chemokine and inflammatory cytokine formation, leading to keratinocyte apoptosis and vessel permeability modifications (31, 32). Finally also some gene polymorphisms responsible for drug metabolism could have a role as risk factors for HFS (31, 32).

In our study, we observed a different prevalence of HFS in sorafenib and lenvatinib groups, with more cases and more severe toxicity under sorafenib therapy, which could probably be explained by the different inhibition potency of the two drugs for VEGFR and PDGFR (Supplementary Table 2).

Concerning lenvatinib therapy, a difference in the frequency and severity of HFS was also present comparing our data with registrative studies and other series in the literature. In particular, Nervo *et al.* observed a quite high frequency of HFS (91.7%) in a cohort of only 12 patients (21). However, in this study lenvatinib represented a second-line therapy in two-thirds of patients; thus, a prior therapy with sorafenib could explain the high prevalence of HFS. Likewise, in the single-center study of Kim S Y *et al.* where sorafenib was used to treat all patients prior to levatinib, HFS was documented in 13/23 cases (56.5%) (19). On the other hand, the study by De Leo *et al*., which included patients treated with lenvatinib as first-line systemic treatment, reported one of the lowest rate of HFS corresponding to 7.7% (33). According to this data, we can hypothesize that the development of HFS and its severity is higher in cases treated with lenvatinib as second line, especially if performed after sorafenib. This was not the case in our series, but the number of patients treated with lenvatinib as second-line treatment was too small to draw any conclusions.

Regarding sorafenib therapy, an higher prevalence of HFS was reported by other authors compared to our results, ranging from 61% to 93.3%, but with a lower severity, since a grade > 3 was found in only 10% per 23.5% (10, 11, 12, 13, 14, 15, 16, 17). The reported prevalence of HFS is similar in studies relating to the use of this drug in other solid tumors. For example, in hepatocellular carcinoma treated with sorafenib HFS was found in 21% of patients with a grade > 3 in only 8% (34), while in renal carcinoma it was observed in 30% of cases with a grade 3–4 in 6% (35). The difference in the prevalence of HFS between different series could be linked to several reasons: a diagnostic difficulty that could lead to an ‘over-diagnosis’ of HFS since there are no specific and selective criteria for this syndrome; previous treatments carried out by the patient which could cause residual toxicity in addition to that induced by sorafenib; different length of follow-up could also explain the different percentage reported as well as TC histotypes, patients’ age and general performance status of patients.

To our knowledge, this is the largest monocentric longitudinal study that includes advanced differentiated and poorly differentiated TC treated with MKIs. The main limitation of the study is its retrospective design, although the data are drawn from a prospectively maintained database.

The risk factors for the development of HFS have been studied extensively in patients receiving sorafenib, being much more clinically relevant than lenvatinib-induced ones. The group of Dranitsaris *et al.* elaborated a predictive model of sorafenib-related HFS composed by female gender, patient performance status of 1 or 2, the presence of liver and lung metastases, two or more organs involved and a normal baseline WBC count (36). They also suggested that cumulative drug exposure, particularly within the first 5 weeks, increased the risk of developing HFS (36). In our study, we did not observe any association with the drug dose but we found that, both in lenvatinib and sorafenib group, the duration of therapy was significantly associated with HFS development. Interestingly, we found that in the lenvatinib group HFS was significantly more frequent in younger patients. One hypothesis could be that younger patients manage to stay on therapy for a longer time because they have a lower number and grade of toxicities. We found indeed a inverse correlation between age and duration of lenvatinib therapy, of low strength but statistically significant. Further studies with larger cohorts of patients are needed to confirm this result.

No international guidelines for the management of skin toxicities are available, but some suggestions could be taken from clinical trials and real-life studies (37). For HFS, it is important to promote preventive measures and proactive managements. The clinician should perform a complete clinical examination before starting the drug, aimed to identifying predisposing factors (hyperkeratosis) or basal lesions (diabetic foot ulcer), that should be treated. The application of keratolytic urea creams might be used to aid exfoliation of calluses, fragrance-free hypoallergenic creams and oily moisturizer could maintain skin hydrated and help to prevent lesions (7, 38). Moreover, in the context of pre-habilitation, patients with advanced TC should be educated to protect feet pressure points and tender areas with comfortable shoes and socks, to avoid contact with water at high temperatures, to recognize HFS first signs, and seek immediate medical attention for early management of HFS. In case of high-grade HFS (G3/G4), in addition to the aforementioned aids, topical therapy for symptomatic pain relief with glucocorticoid and/or analgesic cream could be suggested. In case of unresponsive pain, a glucocorticoid and anti-inflammatory systemic therapy could be used (38). In grade 3–4 HFS clinicians could consider drug dose reduction or temporary suspension. The multidisciplinary management of the patient is fundamental and the main figures involved has to be nurses for medications, clinicians for prevention, medical treatment and drug management and podiatrists not only to provide local medications but also for the preparation of customized footwear in the most severe cases. Whenever possible a preventive intervention of the podiatrists would be desirable.

## Conclusion

HFS is a frequent AE related to lenvatinib and sorafenib intake. While during sorafenib therapy HFS more often presented at a higher grade (grade ≥ 3) and represents the most frequent cause of drug dose reduction, during lenvatinib therapy it was rarely severe to require a drug reduction or discontinuation. Early diagnosis of HFS is particularly important to allow early intervention, possibly in a multidisciplinary setting, and therefore to avoid the discontinuation of treatment, which is highly relevant to obtain the maximum effectiveness of systemic therapy.

Although not scientifically proven, it is likely that a preliminary evaluation with the potential use of therapeutic aids even before starting MKI therapy, the ongoing patient education and a proactive management could reduce the risk of developing HFS and avoid the progression toward higher degrees of severity, that is the reason for dose reductions or suspensions.

## Supplementary Materials

Supplementary Table 1. Relationship between clinicopathological features and duration of MKI therapy in patients with advanced thyroid cancer treated with sorafenib and lenvatinib, respectively.

Supplementary Table 2. IC50 (nM) of the main drugs used in the treatment of advanced thyroid cancer against the membrane receptor involved in skin toxicity.

## Declaration of interest

RE has been a consultant for Bayer for sorafenib development and for EISAI for the lenvatinib one. However, these commitments did not have any influence on this study which has been developed independently and there was no conflict of interest in writing the paper. The other authors declare that there is no conflict of interest that could be perceived as prejudicing the impartiality of the study reported..

## Funding

The study was partially supported by a grant of Ministero dell’Istruzione, dell’Università e della Ricerca Italiano (MIUR, Investigator Grant 2017, PRIN Project, YTWKWH).

## Ethics statement

All patients signed an informed consent to the use of their clinical and biochemical data for research purposes. The present study was approved by the Institutional Review Board.

## Author contribution statement

Study concepts and design: RE; literature research: VC, VC, MV; clinical studies: EMi, VC, LL, CG, LV, LA, EMo, AM; experimental studies/data collection: VC, EMi, MV, AM; statistical analysis: EMi, VC; manuscript preparation: EMi, VC; manuscript editing: RE, EMi, VC.
