# Supplementary material for: Hand–foot syndrome in sorafenib and lenvatinib treatment for advanced thyroid cancer
Source: Eur Thyroid J. 2024 Jul 29;13(4):e240009. doi: 10.1530/ETJ-24-0009 (PMC11301531; doi:10.1530/ETJ-24-0009)
Supplement: Supplementary Table 1. Relationship between clinicopathological features and duration of MKI therapy in patients with advanced thyroid cancer treated with sorafenib and lenvatinib, respectively. [file supplementary_table_1.pdf]

Supplementary Table 1. Relationship between clinicopathological features and duration of MKI therapy in patients with advanced thyroid cancer treated with sorafenib and lenvatinib, respectively.

Variables	N	PANEL A Sorafenib (n=74) Duration Therapy < 1 month (n=7)	1-6 months (n=39)	6-12 months (n=9)	>12 months (n=19)	p	N	PANEL B Lenvatinib (n=165) Duration Therapy < 1 month (n=7)	1-6 months (n=34)	6-12 months (n=33)	>12 months (n=91)	p
**Sex (n, %)**												
Females	40	5 (12.5)	21 (52.5)	4 (10)	10 (25)	0.74	82	4 (4.9)	17 (20.7)	16 (19.5)	45 (54.9)	0.981
Males	34	2 (5.9)	18 (52.9)	5 (14.7)	9 (26.5)		83	3 (3.6)	17 (20.5)	17 (20.5)	46 (55.4)	
**Age at initiation of MKI therapy** (median, IQR, years)		57 (53-67)	61 (53-70)	66 (59.5-72.5)	63 (56-69)	0.66		73 (59-77)	69.5 (62-77.2)	63 (58-71)	64 (58-72)	0.07
**TC histotypes (n, %):**						0.09						0.119
Papillary	21	3 (14.3)	9 (42.9)	2 (9.5)	7 (33.3)		89	3 (3.4)	19 (21.3)	20 (22.5)	47 (52.8)	
Follicular	21	1 (4.8)	9 (42.9)	4 (19)	7 (33.3)		34	0	6 (17.6)	5 (14.7)	23 (67.6)	
Oncocytic	0	0	0	0	0		9	0	1 (11.1)	3 (33.3)	5 (55.6)	
Poorly differentiated	10	3 (30)	4 (40)	1 (10)	2 (20)		30	4 (13.3)	7 (23.3)	3 (10)	16 (53.4)	
Anaplastic	22	0	17 (77.3)	2 (9.1)	3 (13.6)		3	0	1 (2.9)	2 (6.0)	0	
**T stage at diagnosis**						0.46						

**(n, %):**	15	2 (13.3)	9 (60)	1 (6.7)	3 (20)		30	2 (6.6)	8 (26.7)	5 (16.7)	15 (50)	
Tx	2	0	2 (100)	0	0		9	0	4 (44.4)	1 (11.2)	4 (44.4)	0.415
T1	3	0	3 (100)	0	0		30	1 (3.3)	8 (26.7)	4 (13.3)	17 (56.7)	
T2	21	2 (9.5)	7 (33.3)	3 (14.3)	9 (42.9)		61	2 (3.3)	7 (11.5)	14 (22.9)	38 (62.3)	
T3	33	3 (9.1)	19 (54.5)	5 (15.2)	7 (21.2)		35	2 (5.7)	7 (20)	9 (25.7)	17 (48.6)	
T4												
**N stage at diagnosis (n,**							7					
**%):**	1	0	1 (100)	0	0		68	0	0	2 (28.6)	5 (71.4)	
Nx	33	2 (6.1)	17 (51.5)	5 (15.2)	9 (27.3)	0.86	90	4 (5.9)	14 (20.6)	13 (19.1)	37 (54.4)	0.889
N0	40	5 (12.5)	21 (52.5)	4 (10)	10 (25)			3 (3.3)	20 (2.2)	18 (20)	49 (54.5)	
N1												
**M stage at diagnosis (n,**	2											
**%):**	38											
Mx	34	0	1 (50)	0	1 (50)	0.47	13	0	1(7.7)	3 (23.1)	9 (69.2)	
M0		2 (5.3)	19 (50)	7 (18.4)	10 (26.3)		92	2 (2.2)	24 (26.1)	19 (20.7)	47 (51)	0.139
M1		5 (14.7)	19 (55.9)	2 (5.9)	8 (23.5)		60	5 (8.3)	9 (15)	11 (18.3)	35 (58.4)	
**8th edition AJCC Staging**												
I	15	1 (6.6)	10 (66.7)	1 (6.6)	3 (20)		42	1 (2.4)	9 (21.4)	10 (23.8)	22 (52.4)	
II	8	1 (12.5)	1 (12.5)	3 (37.5)	3 (37.5)		36	1 (2.8)	7 (19.4)	7 (19.4)	21 (58.3)	
III	7	2 (28.6)	2 (28.6)	1 (14.2)	2 (28.6)		18	0	4 (22.2)	2 (11.1)	12 (66.7)	
IVA	8	0	4 (50)	2 (25)	2 (25)	0.18	14	0	2 (14.3)	3 (21.4)	9 (64.3)	0.758
IVB	28	3 (10.7)	15 (53.6)	2 (7.1)	8 (28.6)		49	5 (10.2)	9 (18.4)	11 (22.4)	24 (49.0)	
IVC	8	0	7 (87.5)	0	1 (12.5)		0	0	0	0	0	
**Starting dose of MKI** (median, IQR, mg/daily)		-	-	-	-	-		24 [IQR 14-24]	24 [IQR 14-24]	20 [IQR 14-24]	24 [IQR 14-24]	0.154
**Hand-Foot Syndrome**	35						43					
Yes	39	4 (11.4)	12 (34.3)	4 (11.4)	15 (42.9)	0.005	122	0	3 (7)	10 (23.3)	30 (69.8)	0.016
No		3 (7.7)	27 (69.2)	5 (12.8)	4 (10.3)			7 (5.7)	31(25.4)	23 (18.9)	61 (50.0)	
**ECOG PS**	59						72					
0-1	15	7 (11.9)	30 (50.8)	7 (11.9)	15 (25.4)	0.31	50	2 (2.8)	10 (13.9)	19 (26.4)	41 (56.9)	0.906
2-3		2 (13.3)	9 (60.1)	2 (13.3)	2 (13.3)			2 (4)	13 (26)	11 (22)	24 (48)	

Abbreviations: MKI: multikinase inhibitors; TC: thyroid carcinoma; SD: standard deviation; IQR: interquartile range; AJCC: America Joint Committee on Cancer; ECOGPS: Eastern Cooperative Oncology Group Performance Status
