# Supplementary material for: Hand–foot syndrome in sorafenib and lenvatinib treatment for advanced thyroid cancer
Source: Eur Thyroid J. 2024 Jul 29;13(4):e240009. doi: 10.1530/ETJ-24-0009 (PMC11301531; doi:10.1530/ETJ-24-0009)
Supplement: Supplementary Table 2. IC50 (nM) of the main drugs used in the treatment of advanced thyroid cancer against the membrane receptor involved in skin toxicity. [file supplementary_table_2.pdf]

Supplementary Table 2. IC50 (nM) of the main drugs used in the treatment of advanced thyroid cancer against the membrane receptor involved in skin toxicity.

		Molecular targets		
**Drug**	**EGFR**	**PDGFR**	**VEGFR-2**	**Reference**
Vandetanib	0.5	ns	4	30
Sorafenib	ns	57	90	29
Lenvatinib	6500	39	4	28
Cabozantinib	ns	ns	0.03	27

Abbreviations: IC50: half maximal inhibitory concentration; EGFR: epidermal growth factor receptor; PDGFR: platelet-derived growth factor receptor; VEGFR: vascular endothelial growth factor; NS: not significant
